# Lessons From Implementing Ask-Boost-Connect-Discuss, a Peer-Delivered Psychosocial Intervention for Young Mothers Living With HIV in Malawi, Tanzania, Uganda, and Zambia

**DOI:** 10.9745/GHSP-D-23-00077

**Published:** 2023-10-30

**Authors:** Christina Laurenzi, Don Operario, Chipo Mutambo, Eugene Mupakile, Blessings Banda, Fileuka Ngakongwa, Richard Kilonzo, Chuma Busakhwe, Agnes Ronan, Elona Toska

**Affiliations:** a Institute for Life Course Health Research, Department of Global Health, Faculty of Medicine and Health Sciences, Stellenbosch University, Tygerberg, South Africa.; b Department of Behavioral, Social, and Health Education Sciences, Rollins School of Public Health, Emory University, Atlanta, GA, USA.; c Paediatric-Adolescent Treatment Africa, Cape Town, South Africa.; d Paediatric-Adolescent Treatment Africa, Lusaka, Zambia.; e Paediatric-Adolescent Treatment Africa, Lilongwe, Malawi.; f Paediatric-Adolescent Treatment Africa, Dar es Salaam, Tanzania.; g Paediatric-Adolescent Treatment Africa, Kampala, Uganda.; h Centre for Social Science Research, University of Cape Town, Cape Town, South Africa.; i Department of Social Policy and Intervention, Oxford University, Oxford, United Kingdom.

## Abstract

Adolescent girls and young women in sub-Saharan Africa are at high risk of HIV, unintended pregnancy, and early motherhood. These intersecting risks can adversely affect their developmental trajectories and lifelong well-being. Because young mothers living with HIV in these settings experience high levels of stigma, shame, and isolation, tailored psychosocial intervention approaches for this group are critical yet unavailable. Enlisting young peer supporters may be a promising way to expand the reach of health services and enhance psychosocial well-being. To date, few peer-based interventions have targeted young mothers living with HIV. In 2019–2021, we codeveloped a peer-based, facility-embedded intervention package, Ask-Boost-Connect-Discuss (ABCD), with young peer supporters to address the psychosocial needs of young mothers living with HIV in Malawi, Tanzania, Uganda, and Zambia. We then analyzed programmatic data from ABCD to assess the feasibility of using young peers to deliver psychosocial support. Data sources included post-intervention interviews, focus groups, and written feedback from multiple stakeholders (participants, peer supporters, their supervisors, and clinic-based mentors), which were analyzed thematically. We organized our findings according to Bowen et al.'s feasibility framework. Findings spoke to the acceptability, practicality, and integration of the ABCD program. We found that young peer supporters were seen as acceptable program implementers; able to adopt responsive, engaging, and nonjudgmental approaches; and supported through training, technical skills development, and supervision, alongside purposeful facility integration. Importantly, we also found evidence reflecting the roles of demand and adaptation in program delivery (i.e., how peers responded to emerging participant needs or pivoted in their approach based on shifting circumstances). We conclude that considerations of intervention feasibility and/or program fidelity should be attuned to the dynamic qualities of young peer supporters as implementers and should extend beyond standard modes of assessment to consider intervention codevelopment and implementation as an iterative and adaptive process.

## Abstract (precis)

To respond to the distinct needs of young mothers living with HIV, peer supporters can be trained to provide structured psychosocial support. The authors assessed the feasibility of using young peers to deliver this psychosocial support.

## Teaser

**Key Findings:**

- Peer-delivered psychosocial support for young mothers living with HIV can be acceptable, practical, and integrated into existing health structures.
- Importantly, young peer supporters, especially in resource-constrained settings, may be able to contribute as dynamic partners in the cocreation of interventions—not just at the inception phase but as interventions are implemented.
- Young peer supporters working with young people exposed to multiple adversities should receive appropriate training in both content and facilitation skills, as well as ongoing supervisory and debriefing support.

**Key Implication:**

- These findings may be valuable for policymakers, program implementers, and funders seeking to promote integrated service delivery approaches, identify avenues for feasible task-shifting, and maximize program reach and retention for young people facing multiple adversities.

## INTRODUCTION

Young women aged 15–24 years in sub-Saharan Africa are at high risk of HIV, unintended pregnancy, and early motherhood, all of which may adversely affect their mental health and HIV outcomes during this pivotal life stage.^1^^–^^4^ They have the highest HIV incidence of all age groups and are twice as likely to be living with HIV than young men.^5^ Of the estimated 250,000 adolescent girls and young women aged 15–24 years who newly acquired HIV in 2021 globally, 82% live in sub-Saharan Africa.^5^ Additionally, an estimated 20% of girls in sub-Saharan Africa become pregnant by age 19 years.^6^ Learning their HIV-positive status, becoming pregnant, or having a child can be critical inflection points for young women's health.^7^^,^^8^ The dual burden of unintended pregnancy and HIV can lead to social stigma, shame, and isolation.^8^^–^^10^ These experiences may increase distress as young women navigate health care services, education, and employment at a critical time for their health and well-being. They may also compound existing mental health challenges, which have been documented as high across populations of adolescents and young people living with HIV; poorer mental health may increase risks of nonadherence to antiretroviral therapy, disengagement from health care, and risky behaviors linked to sex and substance use.^11^^–^^13^

Because adolescence is a period of developmental potential where lifelong health behaviors are established, it is also a crucial window for intervention.^14^ Broadening access to social support may lead to better care-seeking—improving mental health and HIV outcomes for young mothers and long-term positive health outcomes for their children. However, in resource-limited settings, including much of the African continent, integrated mental health services are limited and mainly focused on clinical mental health treatment rather than broader-based prevention.^11^ Given high levels of internalized and enacted stigma, young mothers living with HIV require early, tailored, sensitive, and nonjudgmental services to support their health and well-being.^15^ Recent systematic reviews found no psychosocial interventions to improve mental health outcomes targeting young women living with HIV^12^ and no interventions for young mothers that have been implemented in low- and middle-income settings.^16^ Moreover, more generalized programming for adolescents living with HIV has not been adapted to support this unique, highly vulnerable group. Ensuring the availability and acceptability of interventions to support this group is urgently needed.

Given high levels of internalized and enacted stigma, young mothers living with HIV require early, tailored, sensitive, and nonjudgmental services to support their health and well-being.

Engaging young peer supporters may be a promising way to deliver nonstigmatizing support to young women living with HIV.^17^ Task-shifting has become a key strategy to manage mental health in low-resource settings where providers are limited and clients' demands are varied and context specific.^18^ Peer-delivered models can reduce burdens on overstretched health systems and increase reach of services at low cost^19^ and have been increasingly piloted to deliver psychosocial interventions for young people living with HIV in low-resource settings.^20^^,^^21^ Trained youth peer facilitators, themselves living with HIV, can provide young people^22^ with more sensitive, supportive approaches to effectively access HIV care.^23^^,^^24^ However, we need more evidence to understand the feasibility of using peers to support the mental health and related health and social outcomes of young pregnant and postpartum women living with HIV.

Feasibility is a central dimension of assessing an intervention at an early stage to determine whether it has the potential to generate significant and sustainable effects. Although guidance for assessing feasibility within an intervention or approach varies across existing implementation frameworks, Bowen et al. identify 8 key areas underpinning the construct of feasibility: acceptability, demand, implementation, practicality, adaptation, integration, expansion, and limited efficacy.^25^ Within these areas, feasibility may be assessed using measures such as fidelity to the intended intervention, quality of implementation, and contextual factors shaping implementation. These domains can also retrospectively guide analysis of intervention implementation data.

In this article, we drew on Bowen et al.'s framework to shape our understanding and assessment of Ask-Boost-Connect-Discuss (ABCD), a peer-delivered psychosocial program to support young pregnant and postpartum women living with HIV.^26^ ABCD aimed to improve the mental health and psychosocial well-being of participants while also upskilling peer supporters. Diverse programmatic evidence can provide critical “real-world” understanding of how programs are implemented and work in practice, shaping future research priorities.^27^^,^^28^ Similarly, lessons from local implementation can shape ideas about how to intervene in broader global settings. We aimed to assess the feasibility of this peer-delivered approach to provide psychosocial support to young pregnant and postpartum women living with HIV. We drew on programmatic evidence from multiple stakeholders across 4 countries who were part of the ABCD program in 2019–2021.

## ABCD PROGRAM

The ABCD program was implemented in partnership with Paediatric-Adolescent Treatment Africa (PATA), a nongovernmental organization working since 2005 to support health care workers and staff, including peer supporters, across 24 sub-Saharan African countries. In 2018–2019, a multisite PATA-research collaboration codesigned and piloted the ABCD program, which was embedded within PATA's existing peer-support program.^29^ PATA's networks of peer supporters comprise young people aged 18–24 years, themselves openly living with HIV, who are trained and mentored to work with young people living with HIV. The peer supporters work in the same communities in which they live, connected to a health facility. Their responsibilities include engaging young people individually and in groups at health facilities, where they are integrated with health teams (clinic-based mentors) and supported by an in-country technical advisor employed by PATA. They also assist with bidirectional referrals between facility- and community-based services and troubleshoot with young people around treatment adherence.

### Design and Training

The ABCD program was codeveloped in 2018 with peer supporters, technical advisors, and a number of young mothers living with HIV themselves, along with a research and implementation team at PATA. The ABCD program aimed to provide peer-delivered group-based psychosocial support to young pregnant and postpartum women living with HIV through 4 domains: “Ask” refers to enrollment and check-ins; “Boost” refers to structured content guiding recurring sessions; “Connect” refers to referral pathways and in-app resources for ongoing self-education for peer supporters; and “Discuss” refers to supervision and case discussion and sharing among peer supporters. Details on the codevelopment and adaptation process are available in a prior linked publication.^26^ Table 1 shows a brief overview of session themes and additional provisions underpinning ABCD.

**TABLE 1.** Overview of Components and Sessions in the Ask-Boost-Connect-Discuss Program to Support Young Mothers Living With HIV

Program Domain	Activity	Description
Ask	Pre-enrollment check-in	Peer supporters used mood charts or verbal prompts to check with participants before they attended group sessions
	Session 0	Welcome session for new participants Physical and emotional health; consent; confidentiality; expectations of participation
Boost	Key components	Structured curriculum with specific content per session; goal-setting worksheets and mood charts linked to each session for participants to take home and use as works for them
	Session 1	Health-seeking behavior Theme: “There are some things I have control over.”
	Session 2	Anxiety and uncertainty in pregnancy and motherhood Theme: “Nobody has all the answers.”
	Session 3	Support networking Theme: “Support can come from different places.”
	Session 4	Self-care during pregnancy and motherhood Theme: “Motherhood is a big change, but I also need to focus on my own well-being.”
	Session 5	Addressing concerns around child growth and development Theme: “Each child grows differently.”
	Session 6	Combating stigma and external judgment Theme: “What others think doesn't matter, if I have love for myself and my child.”
	Session 7	Making time to adopt healthy practices Theme: “Focusing on healthy behaviors takes little effort and is very valuable.”
	Session 8	Future orientation and goal-setting Theme: “Even though I have HIV, I can still embrace a bright future for myself, and my baby.”
Connect	Referral mechanisms for at-risk participants	Set up on a case-by-case basis with clinic-based mentors
	Additional resources for peer supporters	In-app training manual and 1–2 page tip sheets on issues including PMTCT of HIV, mental health psychoeducation, HIV testing, and communication skills
Discuss	Supervision component	Routine supervision meetings by PATA technical advisors; WhatsApp groups formed to facilitate easy communication among peer supporters in each focal country

Abbreviations: PATA, Paediatric-Adolescent Treatment Africa; PMTCT, prevention of mother-to-child transmission.

The Ask-Boost-Connect-Discuss program aimed to provide peer-delivered group-based psychosocial support to young pregnant and postpartum women living with HIV through 4 domains.

The program was embedded in PATA's existing peer support model and designed to be delivered in groups at facilities by peer supporters already engaged in PATA programs. From PATA's larger network, the focal countries (Malawi, Tanzania, Uganda, and Zambia) were chosen for implementation due to their strong existing peer supporter programs and PATA-supported technical advisors. In each country, existing programs were being implemented in a mixture of urban, peri-urban, and rural sites, into which the ABCD program was integrated. Peer supporters received additional training from technical advisors on recruitment and retention of eligible participants; the content of 8 specific modules, which encompassed adapted cognitive-behavioral therapeutic techniques; expectations around intervention delivery and ongoing participant engagement; and referral procedures. Refresher training in communication skills was also completed. Each session consisted of a recap of the prior session theme, introduction of the current session theme, discussion of potential unhelpful thoughts, group brainstorming around helpful thoughts, and group activities to put discussions into practice.^26^

### Program Participants and Implementation

ABCD was specifically geared toward young women aged 18–24 years living with HIV who were either pregnant at recruitment or had young children aged younger than 5 years. Peer supporters and clinic-based mentors were encouraged to engage eligible participants encountered in their routine responsibilities and offer to enroll them into ABCD groups scheduled alongside clinic visits.

ABCD was delivered between February and October 2019 and then extended to be delivered again between January 2021 and November 2021. Content was intended to be implemented in group sessions at 2-week intervals or as dictated by the routine HIV care schedule and attendees' needs at a given facility. Modules were translated onto a basic mobile phone app. Peer supporters received mobile phones from technical advisors with the preloaded app and used the app during sessions to guide the flow of content delivery with participants.

## METHODS

### Study Design

This exploratory study combines multiple method programmatic data collected from diverse stakeholders involved in ABCD program delivery across 4 countries: Malawi, Tanzania, Uganda, and Zambia.

### Sample and Data Sources

Programmatic data were collected from several individuals and sources linked to ABCD implementation in 2019–2021 (Table 2). In brief, data were collected through routine engagements (programmatic check-ins, regular reports, and in-depth follow-up discussions during implementation) with ABCD implementers and stakeholders that used qualitative methods, as well as through additional follow-ups as deemed valuable. Inclusion of ABCD stakeholders in the programmatic data collection was purposive, including the majority of peer supporters involved in ABCD's implementation in all countries, the country-level technical advisors who supported them, and the clinic-based mentors in 1 country (Uganda). Each of these engagements sought to understand the experiences of the pilot ABCD program, focusing on preparation, delivery, content of sessions, and barriers and facilitators. All programmatic data were stored on a secure cloud-based platform with restricted access. Participants gave their written informed consent before any audio-recorded interviews. For additional conversations from which notes were taken, participants gave their verbal consent. Ahead of participation, stakeholders were informed about the confidentiality of their feedback and their personal information; there were also risk mitigation steps taken to ensure that any stakeholder having concerns or experiencing emotional distress could contact the ABCD project manager or be referred to additional community-based resources as appropriate.

**TABLE 2.** Description of Data Sources for the Feasibility Assessment of the Ask-Boost-Connect-Discuss Program to Support Young Mothers Living With HIV

Data Source	Sample Details	Country (Language)	Time Frame	Additional Notes on Data Source
FGDs	Peer supporters (n=5)	Tanzania (Swahili)	September 2019	Translated and transcribed verbatim by in-country technical advisor
	Peer supporters (n=3, 1 per participating facility)	Uganda (English)	September 2019	Summarized in notes format
	FGD1: Peer supporters (n=3), participants (n=2), clinic-based mentors (n=1) FGD2: Peer supporters (n=3), participants (n=1), clinic-based mentors (n=2) i	Zambia (English)	November 2021	Conducted in person, summarized in notes format
Clinic mentor written feedback	Clinic-based mentors (n=3, 1 per participating facility)	Uganda (English)	September 2019	Summarized in notes format
Technical advisor written final reports	Technical advisors (n=4, 1 per country)	Malawi, Tanzania, Uganda, Zambia (English)	December 2019	Received as email correspondence
Technical advisor debriefing session	Technical advisors (n=4, 1 per country)	Malawi, Tanzania, Uganda, Zambia (English)	January 2020	Meeting minutes, from virtual session
Individual semistructured interviews	Peer supporters (n=4)	Malawi, Uganda, Zambia (English)	January 2021	Conducted remotely, audio-recorded, transcribed verbatim

Abbreviation: FGD, focus group discussion.

### Data Analysis

ATLAS.ti software was used to collate, categorize, and code all qualitative data. Open coding was conducted by the first author (CL); the coding framework was reviewed by and discussed with a second member of the research team (ET). Two team members independently coded 2 transcripts, and the full team discussed emergent codes and potential thematic areas (CB, CM). Codes were condensed and themes derived that linked to the implementation of ABCD; emergent themes were discussed with all authors. We used Bowen et al.'s framework to organize our emerging findings and to structure our assessment of feasibility.^25^

### Ethical Approval

Ethical approval for secondary data analysis of programmatic data was granted by the University of Cape Town (Sociology REC2020/10/11).

## RESULTS

Our findings align with 3 key focus areas for feasibility identified by Bowen et al.: acceptability, practicality, and integration (Table 3).^25^ These domains reflected how ABCD is fundamentally acceptable and engaging for young women, deliverable practically through task-shifting approaches, and integrated at the health facility level by design. However, within each domain, we also found more specific examples of peer supporters' ability to tailor and refine content and implementation approaches further, responding to 2 other areas: demand and adaptation. Together, these domains also speak to larger questions around implementation.

**TABLE 3.** Overview of Themes in the Feasibility Assessment of the Ask-Boost-Connect-Discuss Program to Support Young Mothers Living With HIV

Theme	Bowen's Framing of This Domain^25^	Guiding Question in the Context of This Article	Core Theme	Subtheme: Additional Adaptations
Exploring acceptability	To what extent is a new idea, program, process, or measure judged as suitable, satisfying, or attractive to program deliverers? To program recipients?	Is it acceptable to deliver psychosocial content to young mothers and for peers to be the implementers?	Acceptability centered around peer engagement and positive participant feedback	Responding to participant preferences and needs on structure and format
Exploring practicality	To what extent can an idea, program, process, or measure be carried out with intended participants using existing means, resources, and circumstances and without outside intervention?	Is it practical to task-shift to trained peers to deliver a semistructured program to a high standard?	Training, technical competence, and skills development	Responding to emergent needs identified in sessions
Exploring integration	To what extent can a new idea, program, process, or measure be integrated within an existing system?	Can a peer-delivered psychosocial program be effectively embedded within the health system and respond to extant needs innovatively?	Purposeful planning to smooth implementation Challenges to implementation	Adapting to “dis”-integration: pivoting to meet clients' needs in restricted circumstances

### Exploring Acceptability

#### Acceptability Centered Around Peer Engagement and Positive Participant Feedback

Peer supporters and their mentors articulated the challenges young mothers were navigating, which included stigma, social isolation, disclosure, lack of confidence, and perinatal depression and anxiety. The peer status of ABCD's implementers was described as a central feature of its acceptability, enabling participants to feel more comfortable and accepted given these ongoing challenges.

The peer status of ABCD's implementers was described as a central feature of its acceptability, enabling participants to feel more comfortable and accepted given their ongoing challenges.

*We have managed to be close with those young females and hear from them about their challenges, we have managed to make them feel free to talk, even for those who had not shared their challenges before.* —Peer supporter, focus group discussion (FGD), Tanzania

*Young people in the clinic, they have somebody they can talk to. Having someone to talk to is very powerful—people who would not talk to me or a nurse will talk to a peer supporter, [and] peer supporters visit some people in their homes … it's showing that we are on the right track.* —Technical advisor's report, Malawi

This level of comfort was further enhanced by ongoing relationship-building, with a focus on compassion and destigmatization.

*If you don't show them love, some may feel like it's [blaming] them to have a baby or to be pregnant … she can also say, “Oh, maybe the world is judging me, or people judging me for this.” So you have to show them, “No, it's not a thing, no worries,” and we show them that love that they need.* —Peer supporter, Uganda

Peer supporters also shared how cognitive behavioral therapy (CBT)-based approaches encouraged meaningful changes for participants. Across session themes, these approaches were described as useful, applicable, and possible to teach their peers.

*[ABCD] support[ed] them to develop skills, coping strategies and support to change their undesirable situation to desirable to improve their health and well-being.* —Peer supporter, FGD, Uganda

*[CBT] helps them to cope and realize “I have the strength and energy to change the situation.”* —Technical advisor's report, Uganda

Through focusing on smaller, more manageable areas of change, concepts of “de-stressing” and “removing stress” were also emphasized and well received. Speaking about a young mother who had been struggling with stigma while caring for her disabled child, a peer supporter described drawing from ABCD's content to empower her.

*We asked her to avoid self-stigma, not to listen to side words and build courage.* —Peer supporter, FGD, Tanzania

Peer supporters also saw their role in maintaining positive change.

*Some of them, though they know the importance of attending clinic they don't do it without boosting them. So these young women need more attention, because this age group has got more challenges compared to others.* —Peer supporter, FGD, Tanzania

#### Responding to Participant Preferences and Needs on Structure and Format

Although overall acceptability was reported as high, peer supporters made additional adaptations to increase buy-in or respond to specific preferences. Some peer supporters found the session structure and application of CBT approaches repetitive, noting that despite rotating through different topics, it felt like the “same session.” In response, these peer supporters described sometimes using their “own strategies” to introduce the topic and discuss it in another way.

Across sites, the preferred session format also varied. One young Zambian mother described how attending group-based sessions added a crucial social element to her routine antiretroviral therapy pickups. Before joining ABCD, she had received no counseling following her recent HIV diagnosis, and she described feeling lonely, wondering at each pickup, “Is this my life now?” A peer supporter confirmed that participants “were free to open up to each other and also to us peer supporters.”

In the Ugandan site, group engagements were more challenging. One peer supporter ultimately shifted from group to individual engagements to better connect with participants.

*We did 2 group discussions, but they didn't go very well. Most of the time it was me talking and they are just keeping quiet, they are so shy, they don't want to interact. But when it came to one-on-one … there is no rule of shying out, and they can fully be open to you on the one-on-one.* —Peer supporter, Uganda

However, individual engagement instigated further reflection.

*Here they are sharing experiences, and they're like, I've gone through it, I've done this, I've done this, I think maybe some other would be motivated to maybe open up about their issues from there.* —Peer supporter, Uganda

Similarly, a peer supporter in Malawi described a differentiated approach to increase the intervention's acceptability to young mothers.

*The group, some of them were not able to speak, they could just keep quiet the whole session. So, because we are 3 peer supporters [at 1 site], one would find a place and we could tell them, we could do the one-on-ones and then the other ones would be at the group. So, if anyone is having a problem, they would want to see a peer supporter, she could personally come do that, and we meet, and then join the group later.* —Peer supporter, Malawi

In tailoring approaches to better implement sessions, peer supporters used various strategies to engage participants with diverse needs and comfort levels and enhance ABCD's acceptability.

In tailoring approaches to better implement sessions, peer supporters used various strategies to engage participants with diverse needs and comfort levels and enhance ABCD's acceptability.

### Exploring Practicality

#### Training, Technical Competence, and Skills Development

Beyond harnessing peer supporters' “proximity” as fellow young people, ABCD also emerged as practical, through the training of peer supporters and their mentors to ensure a strong command of content, underlying CBT approach, and interpersonal and facilitation skills. Peer supporters detailed how ongoing practice and preparation were important to ensure effective use of time with participants and boost their own confidence and competence in delivering sessions.

*I was confident. Before meeting the young moms, I used to practice.* —Peer supporter, Zambia

*You're also the person that is operating the sessions, you could feel like, you know, sometimes you want to – you need to do research more before you have the sessions with the young mothers.* —Peer supporter, Uganda

*They [peer supporters] are learning from the content. They explore and follow up very intense cases.* —Technical advisor's report, Uganda

Shortfalls in training included limited transparency about how the intervention monitoring was working, leading to questions from 1 peer supporter.

*The training I had, was not that much enough for me … cause, they give us the phones to be using, to record everything. That's why I never knew how they got the information, how it reached them … cause we used to use them but we were sending, so I didn't know if they received the information.* —Peer supporter, Uganda

Another peer supporter described how more training may have been useful; however, peer-mentor and peer-peer relationships also supported implementation.

*Some of the topics were a little bit hard but because the health providers are around, we could call them, so they could help.* —Peer supporter, Malawi

These skills extended to troubleshooting and support-seeking from fellow peer supporters in creative ways. For example, a peer supporter in Malawi spoke about the importance of a WhatsApp group created among a network of peers to share problems and seek input.

Although delivering a mobile phone-guided intervention was new for peer supporters who had only conducted support group sessions without technological assistance, 1 peer supporter noted the value of training on the mobile component.

*Learning how to talk to someone as you're doing something else at the same time—that was a technique that we learned, and right now, we can do it very perfectly.* —Peer supporter, Uganda

However, technical advisors felt that this aspect of communication could be strengthened.

*It distracts the connection [between peer supporter and client], if someone is wanting to say something … connection between facilitator and clients may be disturbed while using the phone. You need to sit there and discuss [an issue] while you have good connection. They just need more training.* —Technical advisor's report, Tanzania

Similarly, additional training to improve peer supporters' counseling and communication skills could practically link to more flexible application of skills in sessions.

*When there is feedback in sessions, they need to know how to deal with this—this is not in the app.* —Technical advisor's report, Uganda

#### Responding to Emergent Needs Identified in Sessions

Peer supporters leveraged their training and close relationships with participants to flag and, at times, respond to emergent topics and needs that fell outside the scope of their training and/or the program sessions.

*We heard that they were pregnant [again], so that's when we later introduced the family planning. But it wasn't even there in the app … it wasn't clearly stated.* —Peer supporter, Uganda

Other peer supporters in Tanzania echoed the importance of family planning content, including supporting health facilities to provide adolescent-responsive sexual and reproductive health services.

*I would like to insist on family planning if ABCD program will proceed … without family planning, we will be doing nothing.* —Peer supporter, FGD, Tanzania

More deeply entrenched structural problems also emerged during sessions, including relationship problems and gender-based violence, which peer supporters struggled to address.

*It is a challenge for young women who have been abandoned by males who impregnated them, they are very depressed because they lack support from their partners, so it was a challenge for them to take care of the pregnancies and the babies. For sure, I always think much about them, but I don't get the right answer.* —Peer supporter, FGD, Tanzania

*There were those topics that, you know, kept coming up … Like for example the GBV, you know, gender-based violence experienced in their homes, that topic wasn't like largely put into the app … it wasn't 1 or 2 young mothers that were facing this kind of violence – because I had like, 14 young mothers? But then 7 of them were facing this problem.* —Peer supporter, Uganda

These challenges highlighted the limitations of ABCD to address broader systemic issues facing young mothers.

*When you look at the app, yes the app would give us guidelines on – we could talk about the situation, but you needed to make [stigma] stop, you needed to make those young mothers feel, you know, free in her life. So the only way to do it is to make it stop, or look beyond what is bringing this all – and stop it.* —Peer supporter, Uganda

These situations showcased peers' ability to effectively identify gaps and consider how ABCD might be practically expanded and tailored based on participant need.

### Exploring Integration

#### Purposeful Planning to Smooth Implementation

Various actors described integration between the clinic and community—distinct from a more conventional approach to health service integration—as critical to implementing ABCD. Peer supporters, embedded in health facilities, were able to effectively engage young mothers during routine antenatal and HIV visits. One clinic-based mentor detailed a positive response to ABCD, noting the roles of multiple stakeholders.

Various actors described integration between the clinic and community—distinct from a more conventional approach to health service integration—as critical to implementing ABCD.

*The clinic staffs and the full participation of the young moms created a positive response by developing [a] clinic approach, which can address mental health and depression cases in the clinic.* —Clinic mentor feedback, Uganda

Technical advisors also described the careful planning that went into structuring the program's training and implementation.

*The selection of the people in the clinic was also carefully thought out … there was no way this peer was going to work alone. It instilled more confidence in the peer because they knew that if there are any challenges, at least the people they trained with would be able to come in and support at any particular moment.* —Technical advisor debriefing

This level of deliberate planning was embedded within the approach to implementation at 1 particular site in Zambia. The health facility team adopted a protocol to enhance ABCD's integration into normal clinic functioning. Peer supporters were introduced to all health workers at this facility as ABCD began and became “part of the scenery,” greeting all expectant women arriving at the maternity ward for antenatal visits to become familiar faces at the facility and destigmatize further interactions. Referral processes were also streamlined—eligible young women living with HIV could be identified by health workers and “fast-tracked” through their clinic visits and subsequently linked with a peer supporter to be recruited for ABCD. Likewise, peer supporters had clear channels for back-referrals to health workers for specific health needs, facilitating smoother, more direct services for young mothers and maximizing integration.

#### Challenges in Integration

In certain cases, poor communication and competing priorities complicated the relationship between peer supporters and facility staff, posing additional challenges for program rollout.

*I faced a big challenge of being not accepted at the facility, it was difficult for the facility nurses and clients themselves to accept me … the way the nurses received me was not that friendly. The second challenge was lack of support from the health provider whom I was assigned to as my supervisor, she did not show cooperation, so she left all responsibilities on me, she only used to tell me, “You go and talk to them,” but she did not support me.* —Peer supporter, FGD, Tanzania

Despite ABCD's embeddedness in health facilities, peer supporters often had to be flexible around meeting spaces due to the “nonclinical” nature of the intervention. Space limitations posed challenges to confidentiality and unencumbered participation.

*I used to sit under a tree behind the PMTCT building, but there was not enough privacy for them to disclose their issues, and they used to say, “We are not that much free to disclose everything because there are [facility] nurses and doctors listening to what we are discussing.” So in encountering that challenge we had to lower our voices like murmuring for people not to hear what we were discussing.* —Peer supporter, FGD, Tanzania

Broader challenges to effective program integration came even when groups were able to meet comfortably; peer supporters faced challenges in ensuring participants' attendance, which could be intermittent due to transport costs, lack of childcare, or the objections of participants' families or husbands to their spending additional time away from home.

*Those young women who live far; they sometimes say that they have to do some home activities before leaving, they have to prepare something for their babies before leaving from home, so it is difficult to attend session on time and sometimes they miss it, so to me it's a challenge because I may prepare a session for a certain number of people but they might not come.* —Peer supporter, FGD, Tanzania

#### Adapting to “Dis”-Integration: Pivoting to Meet Clients in Restricted Circumstances

Pivoting from health facility-based groups, multiple peer supporters suggested home visits as a way to follow up on attendance or check on a participant who had said she would attend. One peer supporter felt that these home visits should become an official part of ABCD.

*I also feel like we should also add home visits … visit them at their homes, we see how they are doing. If like you give them the whole day and be there, to see how things are moving, see what they are doing, what the baby is doing, the whole day – how the mom is handling, yea, things like that. You get a day, you visit 1 mom and see what's done there.* —Peer supporter, Uganda

In Zambia, discussions revealed that peer supporters had started to rotate sessions at the homes of young mothers in well-established groups, at their request, after losing access to a confidential facility-based meeting space.

Notably, through their own initiative, peer supporters further used foundational skills to carry ABCD sessions forward during the early days of the COVID-19-associated lockdowns, although the first iteration of the program had formally ended in October 2019.

*We really felt COVID when it was lockdown— … I myself was … frustrated. I used to think all of those anxiet[ies], you know like, “What's happening tomorrow? What's happening the next day?” I also didn't know what to do. So we then decided, as peer supporters, to go through the app, and see if there was … any help that we could tackle and go on.* —Peer supporter, Uganda

A peer supporter in Uganda described short phone calls that she made to prior participants during Uganda's lockdown.

*It was helping, even if it was just for 30 minutes, or 20 minutes sometimes, it was helping them to come back to their normal self.* —Peer supporter, Uganda

In Malawi, a peer supporter added her experience during COVID-19, noting that her participants were still coming to the clinic for antiretroviral therapy collection and that, despite a hiatus in support group interactions, she was able to help them because she had their information.

## DISCUSSION

To our knowledge, this is the first analysis of real-life implementation data from a multisite peer support program for adolescent mothers living with HIV. In our findings, we saw evidence for the importance of acceptability, practicality, and integration as key criteria to maximize the adaptation and feasibility of the ABCD intervention for multiple delivery sites in sub-Saharan Africa. Session content and CBT-based approaches were found to be acceptable; trained peer supporters, already embedded in health facilities, were able to enhance their skills and become practical partners in task-shifting; and facility-based integration expanded program reach while allowing for routine engagement. Although our findings resonate with domains of Bowen et al.'s conceptual model of intervention feasibility, we elaborate on specific considerations for the delivery of the ABCD intervention in complex real-world—and low-resource—settings.

Adolescent-responsive, nonstigmatizing health services are critical to broader adolescent well-being, and these can include both adolescent-focused content as well as acceptable delivery approaches.^30^ As such, peer-based, facility-embedded programming for young women and young mothers living with HIV may hold promise for addressing psychosocial screening and support and prevention of repeat pregnancy. Additionally, peer-led counseling and mentoring may be an important link between young people living with HIV and the health facility—especially given that mistreatment at clinics has been consistently found to be associated with poor antiretroviral adherence among adolescents.^31^^,^^32^ Moreover, these supportive linkages are particularly critical for adolescent girls living with HIV who become mothers; they may experience multiple adversities in the context of social isolation, meaning they struggle to access health and social services at a time when they most need support.^33^

Adolescent-responsive, nonstigmatizing health services are critical to broader adolescent well-being, and these can include both adolescent-focused content as well as acceptable delivery approaches.

However, more nuanced findings linked to constructs of demand and adaptation provide a slightly more complex picture of feasibility. Examples included the real-time adaptations to program format and structure by peer supporters in response to specific participant needs in “off-script” ways, as well as the need for pivoting while facing health system shifts, most notably during COVID-19 lockdowns. These data also include spaces where peers identified demand but were unable to effectively respond—such as with broader emergent challenges linked to relationships and violence. In reflecting on the feasibility of implementing ABCD (what was actually achieved and/or delivered), it is evident that peers followed many aspects of the intended program plans—applying skills to revisit issues, pivot, and adapt in real time, as well as to confront limitations where shifts were not possible. Thus, although we aimed to explore feasibility linked to the peer-based implementation of a psychosocial support package, the most significant findings were not about content feasibility or fidelity to a package per se but about the dynamic quality of young peer supporters as implementers—their adaptability, flexibility, and varied application of skills as an example of ongoing codevelopment in action.

There is limited evidence that reflects these specific possibilities and tensions within other peer support programs; however, recent findings from a trial in Zimbabwe reiterate how young people may be uniquely positioned to critically assess and respond to emergent challenges while also remaining limited by larger structural or societal issues.^34^ In this trial, community adolescent treatment supporters were trained to provide problem-solving therapy to peers living with HIV in addition to a usual program of peer HIV counseling, while a control group received only peer HIV counseling.^34^ Mental health improved in both groups, with more significant improvements in the problem-solving therapy group. However, the intervention was difficult to implement as intended, especially when identifying actionable plans or goals, because adolescent participants struggled to consider solutions to larger, more entrenched problems that often relied on adult relationships or structural challenges.

Even where youth capacity-building and leadership have been effectively integrated into programming, layered with supervisory support,^35^ it is crucial for peer supporters to be part of ongoing conversations that examine their working and social environments. In addition to reflecting the same “contained” sense of agency that we identified in data from ABCD, these findings also connect to broader debates about how peer-based interventions work (e.g., whether intervention content is a driving influence in effectiveness or whether interpersonal and social support carry more sway in influencing outcomes).^36^^–^^38^ While the field of implementation science—and in programmatic settings, impact evaluations—has focused on indicators such as fidelity and dosage, assessing implementation quality more dynamically could be valuable.^39^ Domains may include implementer competence in “softer” skills around communication and rapport-building,^39^ their responses to emerging obstacles to program delivery, and participants' responses to these pivots. These findings, in particular, can push researchers to be more thoughtful and open to implementation as an evolving process, where goals, processes, or provisions can be re-evaluated and reframed over a project's duration.

### Directions for Further Implementation and Adaptation Research

Responding to gaps that we identified, we believe that some basic provisions could strengthen ABCD and similar programs, as well as advance the research that builds evidence for them, even against the backdrop of broader contextual challenges. While some of these provisions may be obvious, they are not well translated into practice, and so at the most foundational level, our evidence supports a stronger push to actually implement these changes.

First, content within psychosocial programming such as ABCD could be formulated to be more flexible to better address emergent gaps, such as around gender-based violence. Responsiveness to gaps identified in sessions could also mean that peer supporters directly guide improvements and iterations to session content, enabling them to be involved in development as well as implementation. Relatedly, there is a need to develop better protocols guiding how young people are screened into psychosocial interventions and how care or services can be better differentiated to address specific needs of young people living with HIV.^40^ Although screening approaches can be used to effectively help tailor services, individualized approaches may not be necessary or acceptable in all cases. In our formative work codeveloping the ABCD package, peer supporters across all 4 countries cautioned against using mental health screening or a purely individual delivery format, raising the potential for perceived stigma from participants.^26^ However, there is limited research about acceptable modes for screening, including more comprehensive intake assessments, and how these approaches could support better health and well-being outcomes.

Content within psychosocial programming such as ABCD could be formulated to be more flexible to better address emergent gaps, such as around gender-based violence.

Second, to support flexibility in service delivery and enhance peer supporters' confidence in addressing emergent challenges, programming should integrate ongoing training of peers across health care topics and skill sets. Young peer supporters could be considered a workforce-in-development—young people who gain formative skills and who may apply their experience in diverse fields, including health and social care.^41^ Training might also include key information about how intervention monitoring will be done and how peers can be supported to improve their performance in specific areas. Additionally, skills-building and supervision for peer supporters may also include tiered approaches to supervision—such as peer-to-peer sharing and mentorship—to allow for independence and increased autonomy over time.

Third, and crucially, supervision and support systems should be well developed to bolster young implementers. These systems should also link to broader structural considerations and gaps that our findings uncovered—issues that were largely independent from the intervention yet crucial for its success. These include poor relationships with facility staff and lack of meeting spaces, factors identified as barriers in other interventions.^42^^,^^43^ Documenting best practices and spending adequate time on sensitization, planning, and relationship-building might support facility readiness and provider reception ahead of implementation.^44^^,^^45^ Likewise, more robust processes for clinic-community engagement could also facilitate better integration between psychosocial programming and necessary referral systems, including community-based support. Collaborations across health providers, facility leadership, and community structures should be prioritized and built around evidence-informed frameworks. The clinic-community collaboration (C^3^) model, developed by PATA, can guide programming both at the initial set-up phase and as a project takes shape.^46^

On a broader systemic level, we believe our findings also clearly point to the ongoing need to advocate for the recruitment, training, and integration of peer supporters to respond to care gaps in settings with high demand for health services, insufficient resources, and a large population of adolescents and youth. Re-envisioning health systems to be responsive to the needs of adolescents and youth over the long term requires acknowledging the multiple, diverse strengths that peer cadres can offer and identifying a sustainable pathway to better integrate these individuals into health systems.

Finally, these considerations should be necessarily attuned to both the more immediate and broader contexts that young women living with HIV navigate. Demand for psychosocial programming is high and adolescent driven, as young women face continued social and economic flux in the wake of the COVID-19 pandemic.^47^ There have also been reported increases in adolescent pregnancy rates, new HIV infections, and gender-based violence exposure related to health service interruptions and school closures.^48^^,^^49^ Insights from psychosocial programming before and after the COVID-19 pandemic—including how peer-delivered programs adapted in response to COVID-19—may drive future decision-making around how to prepare for additional crises, including pandemics. These decisions may involve tailoring delivery methods to enable remote support or bolstering programs to increase resilience to emerging socioeconomic challenges.^17^

### Limitations

Although we aimed to provide context to the factors and experiences that support a peer-delivered intervention, our findings did not focus on outcomes among ABCD's beneficiaries across sites— young mothers living with HIV. Future work on ABCD would ideally include an evaluation of the program on beneficiaries' mental health, well-being, and HIV outcomes. Additionally, because of our use of programmatic data collected at various stages of the program's delivery, we acknowledge that there may be gaps in site-specific data as well as gaps in our understanding of how certain aspects of implementation played out in specific sites.

## CONCLUSIONS

Our findings make a broader case for codevelopment with youth as an ongoing process, not only an early-stage activity. Task-shifting to peer supporters is feasible and may be a promising strategy to reduce stigma, increase social support, identify specific needs, and expand access to much-needed services for at-risk groups, such as young mothers living with HIV. Considerations of intervention feasibility and/or program fidelity should be attuned to dynamic qualities of young peer supporters as implementers; they should extend beyond standard modes of assessment to consider intervention codevelopment and implementation as an iterative, adaptive process. This approach is also valuable for enhancing peer supporter skills and contributing to future workforce development in countries with growing populations of young people.

## Notes

**Peer Reviewed**

**First published online:** October 17, 2023.
